# Antenatal diagnosis of isolated omphalocele

**DOI:** 10.11604/pamj.2015.21.233.7151

**Published:** 2015-07-31

**Authors:** Safae Lamquami, Nisrine Mamouni, Sanae Errarhay, Chahrazzad Bouchikhi, Abdelaziz Banani

**Affiliations:** 1 Gynaecology and Obstetrics Department I, University Hospital Hassan II, Fez, Morocco

**Keywords:** Omphalocele, abdominal wall defect, perinatal outcome

## Abstract

The concern of obstetric and surgical teams is when diagnosis of omphalocele, the care of the newborn and the prognosis of the malformation, mainly linked to the existence of associated malformations or chromosomal abnormalities. In our case of isolated omphalocele, the overall prognosis was good.

## Introduction

An omphalocele is an abdominal wall defect believed to result from a folding abnormality during development. It has an incidence of 1 in 4000-5000 live births and is characterized by a defect at the umbilicus through which bowel and other abdominal viscera herniate and are covered by a thin membrane consisting of amnion, Wharton's jelly and peritoneum [1]. We report a case of isolated omphalocele and the overall prognosis was good.

## Patient and observation

**Figure 1:**
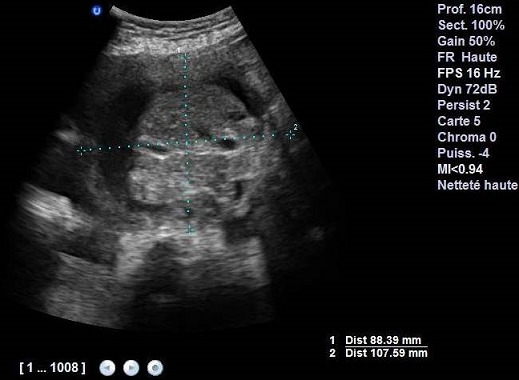
Obstetric ultrasound at 37 weeks containing intestines and part of the liver

**Figure 2:**
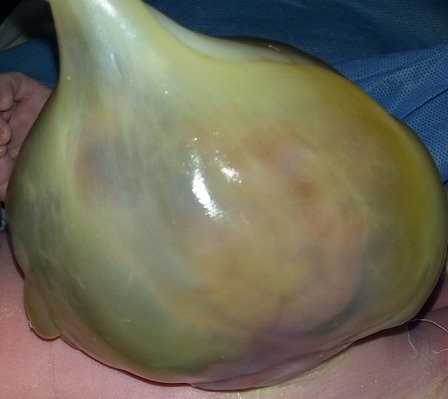
Aspect of the omphalocele at birth

We report the case of a fetus with an isolated omphalocele and whose perinatal outcome was favorable. This is 34 year old woman in labor with no personal or family history. Current pregnancy was followed in our training from 15 weeks of gestation, with discovery of omphalocele prenatally whose diameter was equal 32mm 88 mm at 37 weeks of gestation containing intestines and part of the liver (Figure 1). The assembly is covered by the amniotic membrane, without fetal ascites visualization. No other malformation during the whole pregnancy care (including heart) was detected. The amount of amniotic fluid remained in the standards. A Caesarean was programmed at 39 weeks of gestation. The child was normotrophe, male (Figure 2). The protection of omphalocele in a sterile field was applied immediately and his reinstatement with prosthetic closure was achieved at J1 of life. The karyotype was not asked. The child stayed 72 hours in the neonatology unit without particular metabolic or infectious complications. Currently the infant is 8 months old with good psychomotor development. The surgery to remove plaque is planned soon.

## Discussion

Omphalocele, a congenital defect of the abdominal wall resulting from failure of infolding of the body wall, affects 1 in 4,000 to 10,000 live births and has a higher incidence of 1 in 3000 to 4000 if abortions and stillbirths are considered [1–3]. A other study show that the incidence of fetal omphalocele is estimated as 1:1249 (0.08%) [4]. Abdominal muscles, fascia, and skin are absent; herniated viscera, which can be hollow or solid, are contained in a sac consisting of amnion and peritoneum. Associated anomalies occur in 30% to 70% of cases and chromosomal abnormalities occur in 10% to 30% [2]. It is characterized by the herniation of the abdominal viscera into the base of the umbilical cord, secondary to the failed fusion of the lateral folds during early embryonic development [4]. The incidence of associated anomalies with omphalocele ranged 50-70%, but has been reportedly as high as 80-90% in some studies. The anomalies were not only confined to the gastrointestinal tract. They also involved the heart (up to 40%), neural tube, lip and palate, or cloaca. Some studies suggest that smaller defects (less than 4 cm) are correlated with gastrointestinal defects, whereas larger defects are more likely to be associated with cardiac defects [4]. Although these comorbidities have been well documented, minimal data exist evaluating the presence of systemic hypertension and renal dysfunction in infants with giant omphalocele [1]. As stated in a previous review, chromosomal abnormalities are common. Chromosomal abnormalities particularly aneuploidy such as trisomy 18, 21, or 13 is present in 40-60% of fetuses with omphaloceles not containing liver in 50- 70% of omphalocele cases [4].

Perinatal mortality rate is approximately 30%. Associated anomalies and abnormal karyotype predict increased mortality. Intracorporeal liver and multiple anomalies have been associated with an abnormal karyotype. Paradoxically, both extracorporeal and intracorporeal liver have been associated with improved prognosis by various authors. With improved neonatal care and surgical technique, live-born infants without chromosomal abnormalities or associated severe anomalies have a good prognosis [2]. Omphaloceles are frequently classified as small or giant. A giant omphalocele (GO) is defined as one in which a majority of the liver is contained in the defect. Surgical repair is ultimately required but the method of repair is dependent on surgeon preference, size of the omphalocele defect, associated anomalies and the infant's clinical condition. Options for surgical repair include a primary repair for small omphaloceles in stable infants; a staged closure, such as the Schuster procedure, for stable infants (near term, no respiratory distress) with larger defects assessed to be able to tolerate closure; or escharotic therapy followed by a delayed closure secondary to surgeon preference or in infants that are unstable or with large defects that are assessed to be unable to tolerate a staged procedure in a reasonable length of time [1]. Limited data are available regarding mode of delivery. Available data indicate that cesarean delivery does not improve outcome and may increase maternal morbidity [2]. In isolated cases postnatal outcome depends on type of surgical closure (primary or delayed). Primary closure is defined as closure immediately after birth. Delayed closure is defined as late or even post-infancy closure after allowing the omphalocele todesiccate, contract and epithelializes with closure of the ventral hernia at a later stage. Type of surgical closure is determined by the size of the defect, evisceration of the liver, intra-abdominal pressure, duration of mechanical ventilation, inspiratory oxygen fraction and clinical presentation of pulmonary hypoplasia. Delayed closure is associated with increased co-morbidity and extended hospital stay [5]. The vast majority of the omphaloceles is diagnosed in the first or second trimester of pregnancy. As there are no guidelines on case specific assessment, counseling on individual prognosis is not yet possible, Individual counseling, however, enables parents to anticipate for a relative long period of hospitalization of their infant. We report on additional ultrasound measurements for omphalocele closure which are relevant to the counseling period, i.e. prior to 24 weeks of gestation [5]. To make a definite diagnosis of omphalocele before 12 weeks of gestation, differential diagnosis with physiologic midgut herniation must be made cautiously [4]. Prenatal diagnosis of fetal structural anomalies and growth abnormalities is very important in modern obstetrics. Early diagnosis of fetal structural anomalies and growth abnormalities would make early consultation and management possible, and thus improve maternal and fetal wellbeing. Prenatal diagnosis of fetal omphalocele is a good example [4].

## Conclusion

In cases of ongoing omphalocele, perinatal mortality rates are low in the absence of associated anomalies or genetic defects. Intracorporeal liver was not associated with increased rates of associated anomalies or was it associated with increased neonatal morbidity or mortality [2]. With the advancement and improvement in ultrasound equipment, the early detection of fetal omphalocele is feasible, which will substantially contribute to fetal wellbeing [4].
